# Incidental diagnosis of Mounier–Kuhn syndrome during anesthesia: A case report

**DOI:** 10.1097/MD.0000000000046547

**Published:** 2025-12-19

**Authors:** Biao Feng, Chenxu Sun, Yaping Huang, Yaohui Wang, Hui Yin, Zhihua Sun

**Affiliations:** a Department of Anesthesiology, Xiangya Changde Hospital, Changde, China; b Department of Radiology, Xiangya Changde Hospital, Changde, China; c Department of Anesthesiology, Xiangya Hospital, Central South University, Changsha, China.

**Keywords:** airway management, anesthesia, Mounier–Kuhn syndrome, tracheobronchomegaly

## Abstract

**Rationale:**

Patients with Mounier–Kuhn syndrome (MKS), a rare disorder characterized by tracheobronchomegaly (<0.3% prevalence), develop pathological tracheobronchial dilation secondary to elastic fiber atrophy. MKS predisposes patients to recurrent infections and presents significant airway challenges during general anesthesia. Early radiological recognition is critical to prevent perioperative crises. This case report aims to highlight the critical importance of radiologically identifying MKS preoperatively to avoid potentially life-threatening perioperative airway complications—an aspect that can be overlooked when attention is focused solely on the primary surgical diagnosis.

**Patient concerns:**

A 57-year-old man with multiple risk factors (40 pack-year smoking history, chronic alcohol consumption of >80 g/d, and 10-year betel quid use) was scheduled for partial glossectomy because of suspected tongue cancer recurrence. The patient denied any preoperative respiratory symptoms, and the written report of preoperative imaging revealed bronchitis without tracheobronchomegaly.

**Diagnoses:**

Previously undiagnosed MKS was confirmed intraoperatively and postoperatively. Specifically, postoperative computed tomography revealed tracheobronchomegaly (trachea, 29.3 × 27.3 mm; right main bronchus, 25.5 mm).

**Interventions:**

Following endotracheal intubation under general anesthesia, significant air leakage was observed. Combined with the preoperative computed tomography findings, this raised suspicion for MKS. Precise measurement of the tracheal diameter guided repositioning of the endotracheal tube cuff to 2 cm below the vocal cords, which resolved the air leak and ensured effective ventilation throughout surgery.

**Outcomes:**

Adjusting the endotracheal tube cuff position under imaging guidance effectively resolved the air leak in this patient with MKS, avoiding the need for tube exchange and preventing barotrauma to the fragile tracheal wall from high cuff pressure.

**Lessons:**

For patients with undiagnosed MKS, a comprehensive evaluation of preoperative thoracic imaging is crucial. In MKS, both the cuff placement and size of the endotracheal tube significantly influence airway management success. This is particularly critical in patients with oral cancer, in whom difficult airway conditions reduce the likelihood of successful tube exchange. Optimizing endotracheal tube cuff positioning represents a safe and effective approach. Individualised anesthetic airway management is essential to ensure patient safety.

## 1. Introduction

Mounier–Kuhn syndrome (MKS), first described in 1932 by Mounier–Kuhn, has also been referred to as tracheal diverticulosis or tracheobronchomegaly.^[1]^ It is a rare but clinically significant disorder affecting the central airways, characterized by tracheal diverticula, bronchiectasis, and recurrent lower respiratory tract infections. However, its etiology remains unclear. This case report presents the incidental intraoperative diagnosis of MKS in a patient undergoing oral surgery, during which severe post-intubation air leakage was encountered. This was subsequently managed by endotracheal tube (ETT) repositioning guided by radiographic findings, successfully restoring adequate ventilation. The primary objective of this report is to propose a refined airway management protocol for patients with incidentally diagnosed MKS, who exhibit no preoperative respiratory symptoms and show no tracheal enlargement on preoperative imaging.

Written informed consent was obtained from the patient prior to the submission of this report.

## 2. Case report

A 57-year-old man (height: 170 cm, weight: 52 kg) presented for biopsy and partial glossectomy to evaluate a suspected recurrence of tongue malignancy. Five years earlier, the patient had undergone a radical glossectomy for tongue cancer following a 20-day history of left lingual ulceration and pain. According to anesthesia records from the initial surgery, no airway management difficulties were encountered during induction. Postoperatively, the patient received multiple courses of radiotherapy and chemotherapy. Comorbidities included gout, hypothyroidism, and hypertension. Preoperative evaluation revealed a limited neck range of motion and reduced mouth opening (approximately 2.5 fingerbreadths), attributed to radiation-induced fibrosis. Low-dose chest computed tomography (CT) showed findings consistent with bronchitis and chronic inflammatory changes in the lingular segment of the left upper lung. Notably, during the preoperative anesthetic assessment, excessive reliance was placed on the narrative impressions in the imaging reports (which did not mention tracheobronchomegaly), while direct review of the primary images—including measurement of the tracheal diameter on both CT scans and chest radiographs—was overlooked. The primary anesthetic concern centered on anticipated difficulty with airway management during induction of general anesthesia, exacerbated by anatomical restrictions secondary to previous radiotherapy. Specifically, restricted head and neck mobility, along with reduced oropharyngeal space, posed significant risks to achieving effective ventilation post-induction. Anaesthesia planning prioritized strategies for securing definitive airway access while mitigating the risk of hypoxia.

The patient presented without respiratory symptoms. Upon arrival in the operating theater, oxygen saturation was 98% on room air. Preoxygenation with 100% oxygen was followed by intravenous induction using etomidate 16 mg and sufentanil 15 μg. After loss of consciousness and confirmation of adequate manual ventilation, cisatracurium 12 mg was administered. Because of limited neck extension, nasal intubation was performed using a 6.5-mm internal diameter cuffed ETT (Tuoren Medical Equipment Co., Ltd., Henan Province, China), under direct laryngoscopy with Magill forceps assistance. The tube was secured at 27 cm from the nares. Anaesthesia was maintained with infusions of propofol and remifentanil. Intraoperatively, the anesthesia machine (Dräger Medical AG, Lübeck, Germany) displayed a persistent gas leak, with delivered tidal volumes reduced to approximately 180 mL (set at 400 mL), and end-tidal carbon dioxide elevated to 58 mm Hg. Auscultation revealed diminished bilateral breath sounds, accompanied by an audible air leak from the oral cavity during the expiratory phase of mechanical ventilation. Immediate reassessment of the ETT cuff pressure, the anesthesia machine, and the breathing circuit integrity confirmed normal functionality, with no identifiable technical failures.

**Figure 1.**
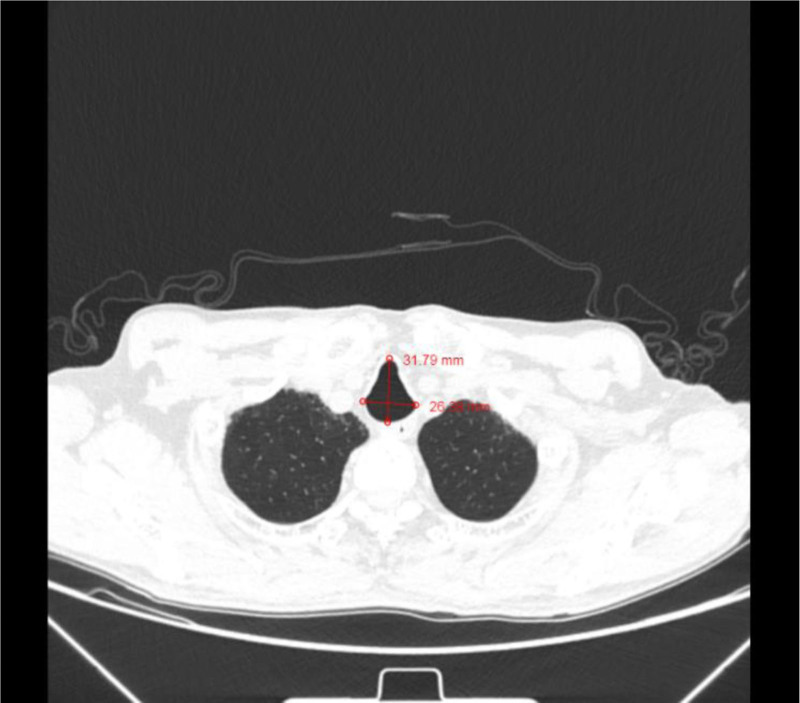
Chest CT imaging demonstrated sagittal and coronal diameters measuring 31.79 and 26.39 mm, respectively, at the maximum transverse cross-section of the tracheal lumen. CT = computed tomography.

**Figure 2.**
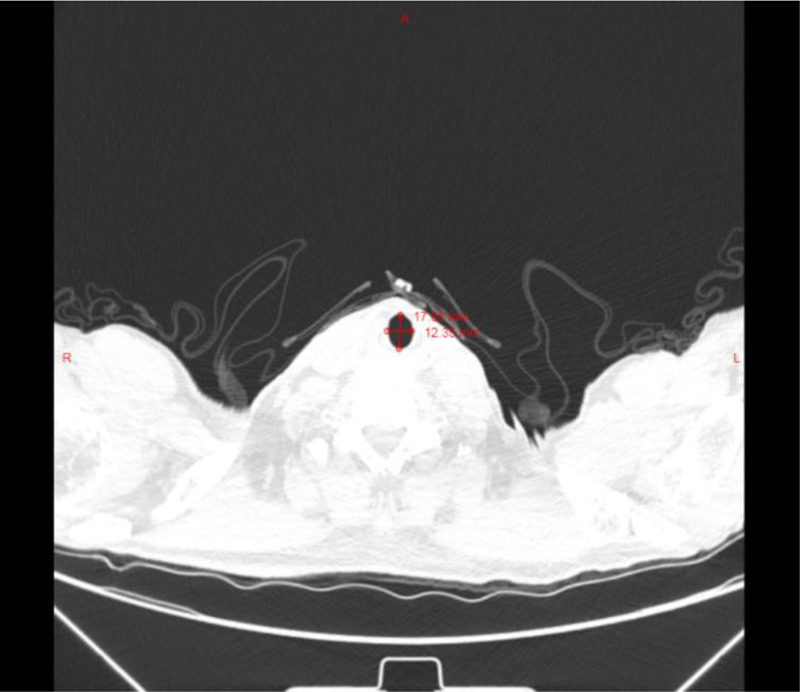
Chest CT imaging demonstrated no significant dilation of the subglottic trachea, with sagittal and coronal diameters measuring 17.05 and 12.35 mm, respectively. CT = computed tomography.

At this juncture, a high index of suspicion for an airway anomaly emerged. Intraoperative evaluation of chest CT images, including precise tracheal diameter measurements, revealed significant airway dilatation. The maximum transverse tracheal dimensions measured 31.79 mm sagittal and 26.39 mm coronal at the level of maximal cross-section (Fig. 1), consistent with diagnostic criteria for MKS.^[2]^ Subglottic tracheal measurements demonstrated sagittal and coronal diameters of 28.1 and 23.6 mm, respectively (Fig. 2).

**Figure 3.**
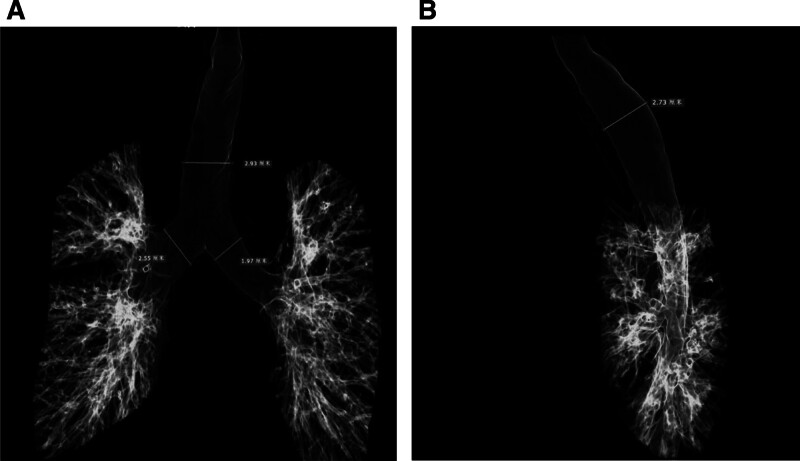
(A) Three-dimensional reconstruction using the CT airway transparency technique demonstrates severe dilatation of the trachea and bronchi. The maximal coronal diameter of the trachea measured 29.3 mm, while the left and right main bronchi exhibited coronal diameters of 19.7 and 25.5 mm, respectively. (B) The maximal sagittal diameter of the trachea reached 27.3 mm. CT = computed tomography.

The observed air leak was attributed to improper cuff positioning within the abnormally dilated tracheal segment. Given the patient’s difficult airway status, history of multiple radiotherapy sessions, and increased risk of oropharyngeal mucosal injury with placement of a larger ETT, we maintained the 6.5-mm internal diameter tube. Quantitative assessment revealed that maximal cuff inflation produced a 24-mm diameter. Under direct visualization with a videolaryngoscope, the ETT was retracted to 25 cm from the nares, achieving optimal cuff positioning immediately subglottic. This maneuver eliminated the air leak and stabilized end-tidal CO_2_ at 40 to 45 mm Hg throughout the 40-minute procedure. Postoperative three-dimensional reconstruction of preoperative chest CT imaging provided detailed bronchial measurements: 19.7 mm for the left main bronchus and 25.5 mm for the right main bronchus in coronal diameter; the maximum sagittal and coronal diameters of the trachea measured 27.3 and 29.3 mm (Fig. 3A, B). These findings confirmed the anatomical airway anomaly and reinforced the diagnosis of MKS.^[2]^ The patient underwent uneventful awake extubation in the postanaesthetic care unit following standard extubation protocols. He was discharged on postoperative day 2 without acute complications during follow-up.

## 3. Discussion

MKS remains a rare clinical entity, with fewer than 20 anesthesia-related cases documented in PubMed to date and no established global consensus on airway management for patients with MKS undergoing general anesthesia. While more than 469 MKS cases have been reported worldwide,^[2]^ advancements in medical imaging and increased utilization of CT scans have led to a 10-fold rise in tracheobronchomalacia diagnoses over the past decade compared with previous decades. A striking male predominance (8:1 male-to-female ratio) characterizes this condition.^[3]^ Preoperative evaluation of MKS presents diagnostic challenges because standard chest radiography often fails to detect tracheal abnormalities and may yield inaccurate tracheal diameter measurements. Chest CT has therefore emerged as the gold standard for MKS diagnosis, enabling precise quantification of tracheal dimensions.^[4]^ Histopathological analysis reveals distinctive features in patients with MKS, including atrophy of elastic fibers, smooth muscle tissue degeneration, and thinning of the muscular layer.^[5]^ The etiology of MKS remains controversial, with ongoing debate regarding its congenital versus acquired origins.^[6]^ Perioperative anesthesia management demands heightened clinical vigilance for the identification of MKS, particularly given the critical need for individualized airway strategies. This includes selecting anesthetic agents with rapid onset and offset to facilitate airway assessment and minimize respiratory depression. Ventilation techniques should be carefully tailored to limit airway pressure and reduce the risk of dynamic airway collapse. This case underscores the indispensable role of preoperative chest CT in both detecting MKS through accurate tracheal diameter measurement and guiding tailored airway management approaches. The integration of advanced imaging modalities into preoperative planning is paramount for optimizing outcomes in this vulnerable patient population.

MKS is primarily a radiological diagnosis, historically established through bronchography^[7]^ and more recently confirmed via CT and X-ray imaging. In the context of modern radiographic techniques such as chest radiography, a tracheal transverse diameter >3 cm serves as a specific diagnostic criterion.^[4,8]^ Spiral CT confirmation requires transverse diameters exceeding 25 mm in males and 21 mm in females.^[4,9]^ Alternative diagnostic parameters include tracheal transverse and sagittal diameters surpassing 25 and 27 mm, respectively, with main bronchial diameters exceeding 18 mm (right) and 21 mm (left) in males, or 17.4 mm (right) and 19.8 mm (left) in females.^[10]^ The diagnostic confirmation in this case report is unequivocal based on these established criteria.

MKS manifests in 3 subtypes: Type I demonstrates mild symmetrical dilation limited to the trachea and main bronchi; Type II exhibits distinctive diverticular morphology; and Type III features extension of these diverticula and saccular structures into the distal bronchi.^[2,11]^ This case represents Type I MKS, where the subglottic region (2 cm below the vocal cords) abruptly transitioned to a markedly dilated main trachea. This anatomical variation resulted in an incomplete tracheal seal by the ETT cuff when positioned within the dilated segment, leading to significant gas leakage that compromised the maintenance of the preset mechanical ventilation tidal volume.

At our institution, the currently available ETT specifications reach a maximum internal diameter of 8.0 mm (Tuoren Medical Equipment Group Co., Ltd., Henan Province, China), with a maximum cuff inflation diameter of 27 mm. This proves inadequate for managing the markedly dilated tracheae seen in patients with MKS. There is an urgent need to develop ETTs with larger cuff capacities, potentially requiring diameters of 28, 30, 35, or even 40 mm. Previous reports have documented patients with MKS who have tracheal lumens exceeding 64 mm in diameter, in which conventional ventilation equipment proved incompatible—necessitating alternative anesthetic approaches such as epidural anesthesia.^[12,13]^

When managing patients with MKS and challenging tracheal anatomy that precludes standard ETT placement, supraglottic airway devices such as laryngeal mask airways have demonstrated clinical utility.^[14]^ Notably, certain patients with MKS present with tracheal dilatation primarily localized near the carina, where the tracheal diameter approximates normal dimensions. In such scenarios, successful surgical outcomes have been achieved by optimizing tracheostomy tube length to accommodate anatomical variations.^[14]^ Recent evidence further supports the efficacy of combining laryngeal mask airways with modified double-lumen Foley catheters as bronchial blockers to facilitate 1-lung ventilation during thoracic procedures in patients with MKS, demonstrating satisfactory intraoperative conditions and favorable postoperative outcomes.^[15]^

The conventional method of using moist gauze packing at the supraglottic level to mitigate air leakage presents particular challenges in oral surgical procedures involving difficult airways. In our case, nasal intubation resulted in significant air leakage, while tube exchange carried substantial risks of failed reintubation and epistaxis. Crucially, CT measurements revealed preserved subglottic tracheal dimensions without significant dilation in this patient. Emerging evidence from thoracic CT and three-dimensional tracheobronchial reconstructions^[16,17]^ identifies a 2-cm segment of relative anatomical preservation below the glottis—a finding that proved fortuitous in our management. The 6.5-mm ETT cuff achieved adequate sealing at this site without requiring excessive inflation pressures.

In the postoperative phase, management should prioritize aggressive airway clearance, infection prevention, and vigilant monitoring for respiratory complications such as atelectasis, pneumonia, and airway obstruction. Early mobilization, chest physiotherapy, and, when indicated, noninvasive ventilation can be beneficial. Extended observation in a monitored setting may be warranted for patients with severe airway involvement or significant comorbidities.

Three-dimensional airway reconstruction in our case demonstrated a fusiform tracheal morphology, underscoring the essential role of advanced imaging in the airway management of MKS. This technology enables precise dimensional analysis of complex tracheal configurations, directly informing both device selection and intubation strategy. Notably, the subglottic zone of anatomical preservation may represent the optimal position for cuff placement in such cases, particularly when managing the disproportionate tracheal dilation patterns characteristic of connective tissue disorders.

In addition to the aforementioned considerations, oral surgical procedures generate substantial volumes of blood and irrigation fluid that may accumulate in the pharyngeal cavity, necessitating exceptional subglottic sealing efficacy to prevent aspiration or tumor seeding. The conventional wet gauze packing method proves suboptimal for oral surgery patients because of the inherent risk of gauze displacement secondary to intraoperative irrigation, which could compromise airway protection. Furthermore, the limited fluid absorption capacity of wet gauze permits potential penetration of blood or irrigation fluids through the packing material into the tracheobronchial tree, thereby increasing the risks of aspiration and potential tumor cell dissemination.

**Figure 4.**
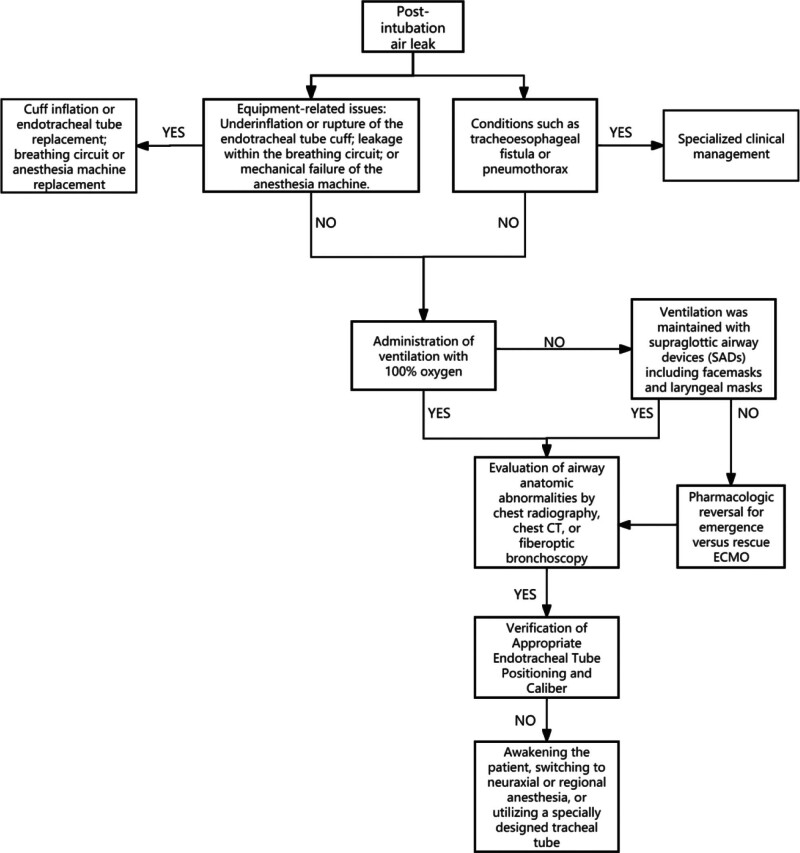
Protocol for managing air leakage after endotracheal intubation.

We have enhanced the existing clinical algorithm for managing post-intubation air leaks in patients undergoing general anesthesia (Fig. 4). This refined protocol integrates recent advancements in airway management technology and clinical experience, potentially serving as a valuable decision-support tool for anesthetists and surgeons across multiple specialties.

Our patient exhibited no preoperative respiratory symptoms; however, inadequate scrutiny of the preoperative chest CT scans initially led to a missed diagnosis of MKS. Although the air leak was promptly recognized and effectively managed following endotracheal intubation under general anaesthesia, this oversight highlights the critical importance of thorough image review. Anaesthesiologists must go beyond narrative report summaries and actively engage in direct evaluation and measurement of radiographic studies during preoperative assessment. In this case, careful reexamination of the preoperative non-contrast chest CT scans ultimately enabled early identification of MKS. For patients undergoing thoracic surgery requiring intraoperative double-lumen endotracheal intubation, precise preoperative measurements of the tracheal and bronchial luminal diameters are essential for appropriate tube size selection. Likewise, in patients scheduled for single-lumen endotracheal intubation, comprehensive review of preoperative chest CT scans or radiographs facilitates the development of an individualized airway management protocol.

The outcomes and insights derived from this case are multifaceted and supported by contemporary literature. This instance highlights the critical importance of comprehensive imaging data for effective intraoperative airway management. Standard chest radiography and conventional chest CT scans typically extend from the lung apex to the base and often fail to adequately visualize the tracheal anatomy. It is particularly important to note that the conventional scanning range from the lung apex (typically at the level of the first thoracic vertebra) to the lung base inherently excludes a critical proximal tracheal segment. Specifically, the glottis (at the level of the cricoid cartilage) is situated at the sixth cervical vertebra, resulting in a gap of approximately 1 vertebral body length between the glottis and the starting point of lung apex imaging, leaving this portion of the trachea unvisualized.While standard chest radiography and conventional chest CT scans—typically extending from the lung apex to the base—may fail to capture the full morphology and internal diameter of the trachea, our patient fortuitously underwent a preoperative neck CT for oral cancer surgery. This extended imaging modality serendipitously provided complete data on the dimensions and configuration of the entire trachea and bronchi, which proved pivotal. It enabled rapid identification and repositioning of the cuff to a radiologically confirmed, non-dilated subglottic segment, successfully resolving the significant air leak. This experience suggests that combined neck and chest CT may offer a more comprehensive preoperative airway assessment, although its routine adoption as a standard requires further consideration. Furthermore, the potential for a catastrophic outcome following an unmanageable air leak underscores the essential need for access to advanced rescue equipment. In such critical situations, extracorporeal membrane oxygenation can serve as a vital salvage therapy, a role increasingly recognized in managing severe airway and cardiopulmonary crises.^[18]^ Ultimately, the entire clinical course—from the initial oversight on preoperative CT to the successful intraoperative rescue—illustrates the indispensable role of the anesthesiologist’s direct and meticulous engagement with preoperative imaging, which forms the foundation for developing safe and individualized airway management strategies.

This study has certain limitations that warrant consideration. Primarily, it is a single-case report that, while offering detailed insights, inherently limits the generalisability of its findings. The management strategies described were effective in our specific clinical setting but may not be directly applicable to all patients with MKS, particularly those with more severe or atypical anatomical variations. Furthermore, as a retrospective analysis of a rare condition, the conclusions are based on the experience of a single institution. The most critical limitation we encountered was the potential for catastrophic outcomes if undiagnosed MKS had led to an unmanageable air leak. In such circumstances, the lack of readily available specialized larger-diameter endotracheal tubes or advanced rescue equipment such as extracorporeal membrane oxygenation could severely compromise patient safety. This underscores the urgent need for improved equipment availability and the development of preformulated contingency plans in clinical settings.

Given the rarity of MKS, airway management in these patients presents unique challenges, where minor oversights may lead to catastrophic outcomes. Clinicians should maintain a high index of suspicion for MKS when encountering post-intubation air leaks, notwithstanding its low prevalence. This case underscores the imperative for thorough preoperative radiographic assessment to formulate optimized anesthetic strategies.

## Author contributions

**Investigation:** Yaohui Wang.

**Resources:** Hui Yin.

**Supervision:** Yaping Huang.

**Software:** Hui Yin.

**Writing – original draft:** Biao Feng, Zhihua Sun, Chenxu Sun.

**Writing – review & editing:** Biao Feng, Zhihua Sun, Chenxu Sun.
